# Blood pH Changes in Dental Pulp of Patients with Pulpitis

**DOI:** 10.3390/diagnostics14111128

**Published:** 2024-05-29

**Authors:** Pedram Hosseinzadehfard, Neringa Skučaitė, Vita Maciulskiene-Visockiene, Greta Lodiene

**Affiliations:** Department of Dental & Oral Pathology, Faculty of Odontology, Academy of Medicine, Lithuanian University of Health Sciences, Eiveniu g.2, 50009 Kaunas, Lithuania

**Keywords:** pulp inflammation, pulpal blood, pH, pH meter, diagnostics

## Abstract

The severity of pulpitis is a crucial factor in determining the suitable treatment. There are no clear objective indicators to assess the stage of pulp inflammation that could be used in clinical practice. The change in blood pH of the pulp during the inflammatory phase could hypothetically serve as an indicator of the pulp inflammation severity. The aim of this study was to assess the pH values in the pulpal blood of mature teeth in patients with symptomatic pulpitis, in comparison with the healthy controls. The study included patients with symptoms of pulpitis in premolar or molar teeth (Test group; *n* = 24, mean age 36.04, SD 7.10), and healthy controls (Control group, *n* = 6, mean age 24.5, SD 5.94) referred for extraction of premolars or third molars, for orthodontic reasons. The pulpal blood was taken at the opening of the endodontic access cavity, and the pH value was measured in both groups. Statistical analysis was performed using the SPSS 27.0 program with a significance level of *p* ≤ 0.05. The Mann–Whitney test for dependent samples was performed to evaluate the statistical difference between the groups. The patients with symptomatic pulpitis had significantly decreased pulpal blood pH compared to the healthy controls (*p* ≤ 0.05). The mean pulpal blood pH was 7.27 (SD 0.06) and 7.40 (SD 0.02) in Test and Control groups, respectively. In the Test group, the pulpal blood pH values were significantly lower in the patients who had symptoms for 3 days or more (7.25, SD 0.05) than in those who had symptoms for up to 3 days (7.33, SD 0.03) (*p* < 0.001). The pH value of the pulpal blood gradually declines in cases of symptomatic pulpitis, along with increasing duration of the symptoms.

## 1. Introduction

Pulpitis is a dental disease marked by an inflammatory reaction of the pulp tissue, usually caused by a bacterial infection [1]. Traditionally, the conventional treatment method of pulpitis has been complete pulp removal followed by root canal treatment (RCT). However, the interest of researchers and clinicians in less-invasive treatment techniques aiming to preserve partial vitality of the pulp has notably increased in recent years [2]. Previous studies have shown that in cases of symptomatic irreversible pulpitis, the tissue located in the coronal pulp chamber is affected by the inflammation, while the radicular pulp is still viable [3,4]. In such cases, removal of the coronal part of the pulp (pulpotomy) allows the maintenance of blood flow in the remaining pulp tissue that could heal and recover [5]. Considering the well-proved healing potential of the pulpal tissue, it has been suggested that recognition of the severity stage of pulpitis (initial, mild, moderate, or severe) can play a major role in selecting the appropriate therapy [3].

The diagnosis of pulp inflammation relies on the clinical symptoms (pain) as well as on the data obtained from subjective (response to thermal and electrical stimulus) and objective pulp vitality (pulse oximetry, laser Doppler flowmetry) tests [6]. However, pain is not always present in cases of pulpitis, and the accuracy of thermal or electrical tests is questionable [7,8].

The limitations of the subjective diagnostic tests in accurately reflecting the true condition of the pulp have fueled the exploration of novel methodologies. In response to the challenges shown by patient-dependent and unpredictable diagnostic tests, a range of non-invasive and true vitality tests has been evolved, including thermography, transmitted light photoplethysmography, laser Doppler flowmetry, and pulse oximetry [9]. Most of them are still under investigation and, despite some promising results, their clinical application is still very limited [8,10].

A number of studies demonstrated that irreversible pulpitis was associated with different expressions of various biomarkers [11,12]. During the inflammation process, granulocytes secrete enzymes in the place where the tissue breakdown starts. High levels of these enzymes could indicate the pulp inflammation stage [13]. A review conducted by Duncan et al. (2022) found that there were increased levels of various mediators, such as interleukin (IL)-1β, IL-2, IL-6, IL-8, and tumor necrosis factor alpha (TNF-α) in samples of irreversible pulpitis compared to healthy pulp [14]. Another potential indicator of tissue degradation in the inflamed dental pulp is the activity of matrix metalloproteinases [15,16]. A few studies reported that the Matrix Metalloproteinase-9 (MMP-9) amount in the damaged pulp tissue was significantly higher than in the healthy counterparts [15] and increased with the severity of pulp inflammation [16]. Thus, the estimate of MMP-9 concentration could potentially serve as an objective indicator of the clinical pulp inflammation stage [16]. However, the clinical evidence is insufficient, and further studies are needed to provide more substantial results.

The mechanism of pH changes in the inflamed pulp has not been thoroughly investigated. It has been proposed that the blood pH of the inflamed pulp is reduced as compared to the blood pH of the healthy pulp [17]. The inflammatory acidosis could develop due to formation of a hypoxic environment in the pulp, as determined by the specific tooth anatomy. Although the pulp is extensively supplied with blood, the main blood vessels enter through a narrow apical opening, there is little or no collateral circulation, and the surrounding hard tissues are rigid [18,19]. For these reasons, the pulp tissue is very sensitive to inflammatory stress, which leads to local ischemia in the pulp tissue during inflammation [19].

An experimental study with rats provided estimates of the pulpal blood pH in artificially induced inflammation [20] and suggested that the pulpal blood pH changes could serve as an indicator for the severity of inflammation in the pulp tissue. The information about the blood pH changes in human pulp is very limited. In a study of Shmueli et al. (2020), the acidity levels of the exposed pulp were estimated in the primary molars with deep caries lesions, with no clinical symptoms of the pulp inflammation using the color of the exposed blood and the time to achieve hemostasis as the determining factor for the following treatment strategy [21]. The estimated blood pH values of the dental pulps subjected to pulpotomy and pulpectomy were similar and did not provide an answer about the acidity levels in the pulp of teeth. No reports about the pulpal blood pH changes in the permanent teeth were identified by the authors of the present study.

Thus, the aim of this study was to assess the pH values in the pulpal blood of mature teeth in patients with symptomatic pulpitis and compare them with the pH values obtained in healthy teeth.

The null hypothesis for this study is that there is no significant difference in the pH levels of pulpal blood between individuals with symptomatic pulpitis and those with healthy dental pulp.

## 2. Materials and Methods

### 2.1. Study Population

This clinical research was approved by the Bioethics Center of the Lithuanian University of Health Sciences (code number is: BEC-OF-142).

The unit of investigation was a tooth. The sample size was calculated using the minimum number of teeth to state the difference between the mean values of the studied variables. Based on Shmueli et al. (2020), the mean value for inflamed pulpal blood pH was 7.52, and for healthy blood, it was 7.40 [21,22]. By considering an alpha error = 0.05 and power of 80%, and based on the following formula, the sample size was estimated to be 14 teeth in total.

**Figure 1:**
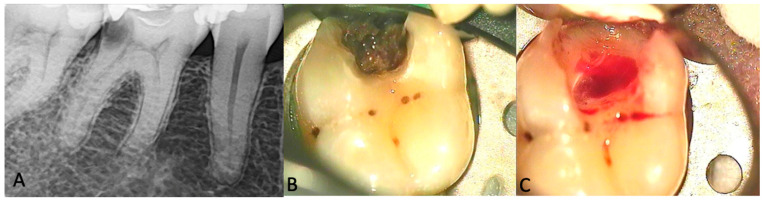
Moderate/Severe pulpitis of tooth 46. (**A**)—Radiographic examination, (**B**)—Clinical aspect after removing old restoration, (**C**)—After cleaning of cariogenic tissues, extensive bleeding from pulp chamber can be seen.

Patients referred to the Clinic of Dental and Oral Pathology, the hospital of Lithuanian University of Health Sciences, Kaunas, Lithuania or to the private dental clinic and diagnosed with symptomatic pulpitis were considered as potential candidates for the study. Only premolar and molar teeth were included to obtain comparable amounts of pulp tissue. The mean age of the patients included in the Test group was 36.04 (SD 7.10), while the mean age of the Control group patients was 24.5 (SD 5.94). The diagnosis was confirmed based on the clinical and radiographic criteria: spontaneous pain and/or lingering pain in response to a thermal stimulus, and no radiographic evidence of the periapical or other endodontic pathology (Figure 1A). Cases with resorptions, calcifications, unrestorable crowns or negative response to the cold test were excluded. Medically compromised patients (with immunosuppressive/systemic diseases, patients on medications) and those who used non-steroidal anti-inflammatory drugs (NSAIDs) before the treatment, as well as patients unwilling to participate, were excluded from the study. Twenty-four patients were selected for the Test group. Both groups were given oral and written information about the study design and signed informed consent forms before treatment. Moreover, they had the right to withdraw their participation from the study at any time. The Test group included patients with **moderate pulpitis** (characterized by a strong, heightened, and prolonged reaction to cold that can last for minutes, possibly with percussion sensitivity and spontaneous dull pain that can be somewhat relieved with pain medication) and **severe pulpitis** (marked by spontaneous pain and a clear pain reaction to warm and cold stimuli, often sharp to dull throbbing pain, difficulty sleeping due to increased pain when lying down, and extreme sensitivity to touch and percussion) [3]. The duration of the symptoms, counting the days from the first symptoms, was registered. The Control group included 6 patients with clinically healthy third molars or premolars that required extraction due to orthodontic reasons; the teeth had no clinical signs of pulpitis, no caries, no restorations; a normal response to the cold test, and had no apical radiolucency on radiographs.

### 2.2. Clinical Procedures

All clinical procedures (except for tooth extraction in the Control group) were performed by a postgraduate endodontics resident (PH).

**Figure 2:**
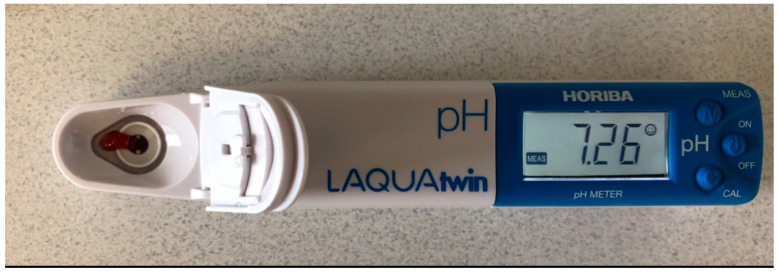
Pulpal blood measurement using Horiba Laquatwin pH-33.

Test group: Under local anesthesia (Articaine 4% Ubistesin Forte 1.7 mL N50—3M ESPE Dental AG; Seefeld, Germany), the tooth was isolated using a rubber dam system, and endodontic treatment was initiated (Figure 1B). The endo access cavity was prepared with a high-speed diamond bur with water coolant. After the pulp chamber was penetrated, excess bleeding was observed (Figure 1C), and the pulpal blood was collected using a sterile 21-gauge needle (Zarys, dicoNEX Co., Zabrze, Poland) from the coronal pulp. The pulpal blood pH was determined using a Horiba Laquatwin pH-33 measuring device (HORIBA Advanced Techno Co., Ltd., Kyoto, Japan) (Figure 2). The subsequent endodontic treatment was performed according to the recommendations from Wolters et al. [3], either pulpotomy or pulpectomy, depending on the symptoms (moderate or severe pulpitis), and for the pulp bleeding control.

Control group: Under local anesthesia (Articaine 4% Ubistesin Forte 1.7 mL, 3M ESPE Dental AG; Seefeld, Germany) the endo access cavity was prepared, and the pulpal blood pH was determined by applying the procedures mentioned above. Thereafter, the extraction procedure was conducted.

### 2.3. Statistical Analysis

Statistical analysis was conducted using the SPSS 27.0 program with a significance level of *p* ≤ 0.05. Means, percentages, standard deviations (SD), median, mode, inter-quartile range, and minimum and maximum values were calculated. The normality of the distribution of the quantitative variables was assessed using the Kolmogorov–Smirnov test. Pearson correlation, Mann–Whitney, and Kolmogorov–Smirnov tests for dependent samples were conducted. The Mann–Whitney test was used for a non-parametric quantitative comparison of variables between two independent groups.

## 3. Results

**Figure 3:**
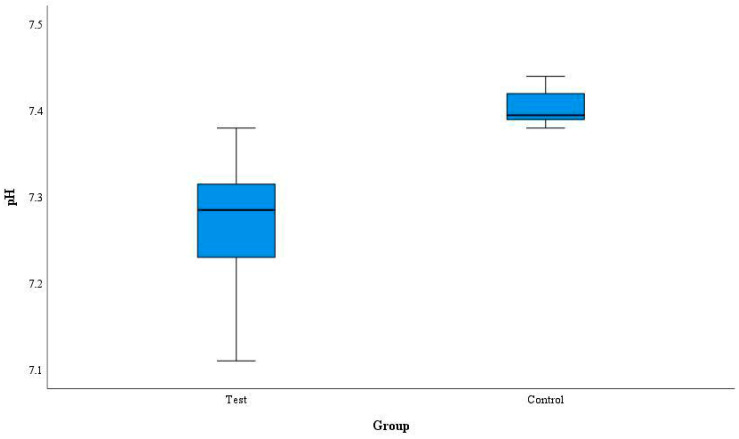
Pulpal blood pH in healthy and inflamed pulp.

The patients with symptomatic pulpitis had significantly lower pulpal blood pH in comparison to the healthy ones (*p* < 0.001). The estimated mean pulpal blood pH value was 7.27 (SD 0.06) for the Test group (*n* = 24) and 7.40 (SD 0.02) for the Control group (*n* = 6), respectively (Figure 3). Both groups included premolar or molar teeth.

The pH values of the pulpal blood were in the ranges of 7.11–7.38 and 7.38–7.44 in cases of moderate or severe pulpitis and in the healthy pulp, respectively. The mode of the pulpal blood pH values estimated in the Test group was 7.27.

**Figure 4:**
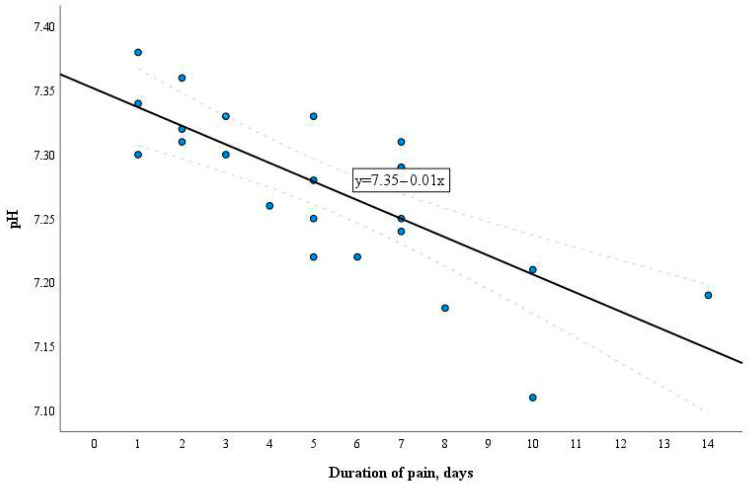
Correlation of the pulpal blood pH level and the duration of symptoms.

The pH levels of the pulpal blood were evaluated according to the duration of symptoms in the patients with moderate or severe pulpitis, and the correlation between these parameters was assessed (Figure 4).

**Figure 5:**
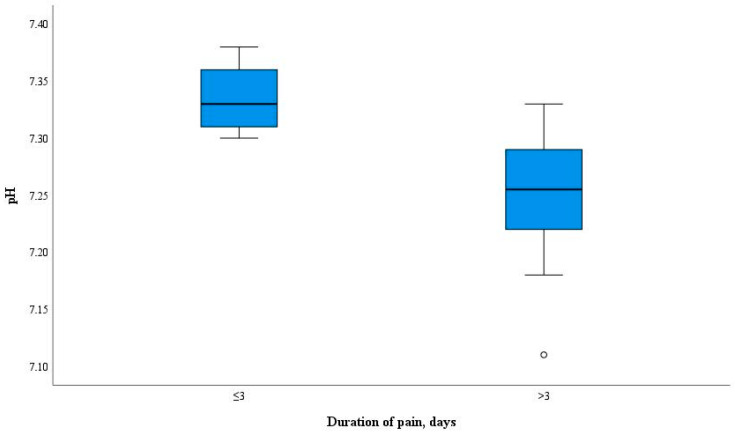
Pulpal blood pH range with respect to duration of symptoms.

With the prolongation of the symptomatic period, there was a decrease in the pH level in pulpal blood. There was a statistically significant difference between the mean pulpal blood pH value calculated in the samples of the patients with symptoms lasting less than 3 days (*n* = 6) compared with the mean value in those of patients with symptoms lasting 3 or more days (*n* = 18), of 7.33 (SD 0.03) and 7.25 (SD 0.05), respectively (*p* < 0.001). The pH values of the pulpal blood ranged from 7.31 to 7.36 when symptoms were less than 3 days and from 7.23 to 7.27 when the symptoms lasted 3 or more days (Figure 5).

## 4. Discussion

Accurate clinical diagnosis is the main concern in the treatment process for pulp inflammation, particularly when the minimally invasive disease management approach is acknowledged. In cases of irreversible pulpitis, the treatment choice between pulpotomy and RCT depends on the degree of the inflamed tissue [23]. Following the classification proposed by Wolters et al. 2017, coronal/partial pulpotomy or RCT are the recommended treatment options in cases of moderate and severe pulpitis [3]. It is evident that the stage of pulp inflammation can be estimated histologically [24]. However, histological evaluation is not possible in clinical practice where the clinician should decide regarding the treatment method either before or during management of the pulpitis. Moreover, the correlation between clinical symptoms and pathological pictures of the pulp status is still arguable [17,24]. In particular, the intensity and duration of bleeding from the inflamed pulp were considered important for determination of the disease severity stage, suggesting various time limits needed for hemostasis [5,25]. However, no evidence has been provided on whether the amount or duration of bleeding could be used to predict the outcome of pulpotomy [26]. On the contrary, there are studies suggesting that the bleeding control of the exposed pulp does not provide a reliable assessment of the inflammation and may be misleading with regard to the chosen treatment strategy [27]. More results are expected to come from several clinical trials designed to investigate the long-term success of pulpotomy in teeth with irreversible pulpitis following the strictly defined protocol, including achievement of complete hemostasis in 10 min [28]. In parallel, research should focus on other potential and more accurate tests that could provide an estimate of the degree of pulp inflammation and aid in defining the limits for the successful outcomes of pulpotomy.

It is well known that inflammatory processes and bacterial infection are associated with acidosis, as outlined in the introduction of this manuscript. The bacteria, the major etiological factor in pulpitis, release cytokines, mediators, and other substances to promote inflammation. In clinical practice, no biomarker test has shown superior clinical and analytical performance. IL-6 and IL-8 have been shown to be reliable biomarkers for diagnosis of irreversible pulpitis; however, the inability to discriminate between reversible and irreversible pulpitis is a major limitation [12]. These compounds can reduce pulpal blood pH and elicit an immune response [23,29]. Therefore, the estimate of the pH change in the pulpal blood could potentially serve as an indicator of progression of the inflammatory process occurring in the pulp, provided it is easily applicable in clinical settings.

In the present study, the Horiba Laquatwin pH-33 handheld device (HORIBA Advanced Techno Co. Ltd., Kyoto, Japan) was selected as a rapid and practical method of measuring the blood pH. This device’s portability and simplicity of use are advantages, enabling all readings to be made in a clinical setting. It also offers a digital readout that does away with the necessity for manual data analysis. The pH meter’s sensitivity of up to 0.01 pH is extremely precise, which was crucial in the present study. The glass electrode technique employs two electrodes, namely a glass electrode and a reference electrode. It gauges the pH of a solution by assessing the voltage (potential) existing between these electrodes. This approach stands as the predominant choice for pH measurement due to its rapid attainment of equilibrium, consistent reproducibility, and ability of application to diverse solution types. Furthermore, the influence of oxidizing or reducing substances on the results is minimal [30].

However, there are certain restrictions with the pulpal blood pH measurements related to the method itself as well as to interpretation of their results. Thus, taking out the blood from the pulp chamber can be challenging. A 21-gauge needle that could fit for the extraction of blood was used in this study. Also, it can be challenging to collect a sufficient amount of pulpal blood for a pH test in some clinical cases since the processes of calcification and necrosis taking place during pulp inflammation may considerably reduce the amount of pulpal blood.

When measuring the pulpal blood pH, it is crucial to avoid sample contamination with saliva to preclude erroneous results. As shown previously, the presence of a periodontal pathology can alter the pH levels of saliva [31,32]. Therefore, to prevent contamination and achieve precise pH measurements, application of an isolation system such as a rubber dam is required.

Moreover, research has demonstrated that different variables independently can regulate the blood pH [33]. Numerous illnesses disrupt metabolic processes in the body, leading to either acidity or alkalinity. For instance, diabetes and acidosis both result in insulin resistance, which in turn leads to acidity [33,34]. Therefore, the general health status of the patient needs to be considered when performing the pulpal blood pH measurements. It is not clear whether the presence of general illnesses can significantly alter the diagnostic potential of the pulpal blood pH measurements, in cases of pulpitis.

According to different studies, the blood has a pH in the range of 7.35 to 7.45 [22]. The pH of healthy human dental pulp tissue has been reported to be around 7.4 [35]. In the present study, the values of the pulpal blood pH in teeth without clinically determined inflammation varied between 7.38 and 7.44. Our findings support the evidence that infection and inflammation are linked to the acidic pH of the human tissues [36,37]. When compared to the healthy pulp, the pulpal blood pH in cases of pulpitis was estimated to be more acidic, ranging from 7.11 to 7.38. The findings of our study suggest that since the highest pH value of the inflamed pulp was evaluated at 7.38, this value served as the “breakpoint” between the healthy and inflamed pulp tissues. More studies are needed to determine the validity of this “breakpoint”. The pH levels of the pulpal blood in cases of symptomatic pulpitis have not been studied before; hence, there are no published data in the scientific literature to compare with the findings of the present study. Thus, it could be suggested that the pulpal blood pH values measured in the inflamed pulp in the present study could inspire future investigations of the pulpal blood pH changes as a potential tool enabling preservation of the uninflamed parts of the pulp and promoting a conservative treatment approach. Furthermore, to disclose if the pH value of the pulpal blood could serve as a possible biomarker of the inflammation degree (mild, moderate, severe), correlations between the measurements of the pulpal blood pH and the outcomes of the vital pulp treatment should be investigated.

The duration of the symptoms typical for moderate/severe pulpitis was recorded when selecting the patients for this study as well. As reported earlier, the stronger the pain, the more advanced is the histopathogenesis of inflammation in the pulp [38,39,40]. Animal studies have shown that once the dental pulp is exposed to oral bacteria, the development of pulp necrosis and, thereafter, periapical periodontitis can be rapid. Generally, this process has been shown to evolve within a few weeks [41]. It is important to note that the pathogenesis of the disease primarily depends on the duration of microbial irritation. An animal study conducted by Renard et al. 2016, examined the histological and molecular response to pulpal injury and infection by artificially exposing rat pulps to phosphate-buffered saline or lipopolysaccharides. The tissues were examined at 3 h, 9 h, and 3 days after the exposure. Pulps in the intact teeth were used as controls. Infiltration by inflammatory cells was seen as early as 9 h after the exposure, and an osteodentine matrix was noted 3 days after the exposure. In the same study, it was also found that MMP3 was upregulated after 3 days of exposure [42]. The MMP-3 production during pulp tissue inflammation stimulates degradation of the surrounding collagen, leading to changes in extracellular matrix structure, inflammation and angiogenesis [43].

In the rat experiment with exposed dental pulp, the initial reaction in the periapical tissues to a clear pulp exposure involved an increase in polymorphonuclear leukocytes (PMNs) and monocyte cells between days 0 and 3 after the exposure [44].

It is well known that the dental pulp and the periapical tissues undergo inflammatory changes during bacterial invasion, although the time frames between reversible to irreversible pulpitis are not yet researched. A correlation has been found between the degree of pulpal inflammation and the amount of bacterial irritants and their proximity to the pulp; however, it can be a challenging task to determine the threshold when the inflamed dental pulp goes from reversable and enters the irreversible category of pulpitis [45,46]. Therefore, evaluation of the acidity levels of the pulpal blood could serve as a potentially good method, facilitating the clinical decision to preserve pulp vitality and avoid root canal treatment.

The histological typing of inflammation illustrates the early stages of pulpitis, showing infiltration of neutrophils mixed with lymphocytes, plasma cells, and eosinophils. In the later stages, infiltration of lymphocytes, macrophages, and plasma cells takes place [23].

In the present study, the time frames were selected in accordance with research demonstrating that acute inflammation might be seen in the first 3 days following the onset of symptoms and progress to chronic inflammation or even necrosis in the following 3 days [23,47]. The pulpal blood pH measurements in the Test group correlated with the duration of clinical symptoms. The results of this study demonstrated that the level of pH in the pulpal blood drops as the period with clinical symptoms continues. The pulpal blood pH values in the patients with symptoms for less than 3 days were equal to 7.31 and higher, while in the patients with symptoms lasting 3 days and longer, the pH values did not rise over 7.27. This finding indicates that evaluation of the pH changes in the pulpal blood might be useful for differentiation of the inflammation severity and consequently, for the choice of the most optimal treatment strategy. More research is needed to validate the pH changes related to the inflammation stages (mild, moderate, severe).

The present study considered the pH changes in a small group of patients with symptoms of pulpitis. Nevertheless, a significant difference was found between the blood pH values of the healthy and inflamed pulp. Thus, the null hypothesis was rejected.

The small sample size certainly limits the generalizability of the obtained data. Investigations using more extensive samples as well validation of the method in a clinical setting are needed prior to applying this method in clinical practice.

## 5. Conclusions

The blood pH of the pulp decreases in cases of symptomatic pulpitis and is related to the duration of clinical symptoms.

## Data Availability

The data presented in this study are available in this published article.
